# The effect of *Zataria multiflora* extract on the clinical endometritis and reproductive indices in lactating Holstein dairy cows

**Published:** 2016-12-15

**Authors:** Abolfazl Hajibemani, Abdolah Mirzaei, Abbas Rowshan Ghasrodashti, Mohammad Reza Memarzadeh

**Affiliations:** 1 PhD Candidate, Department of Clinical Sciences, School of Veterinary Medicine, Shiraz University, Shiraz, Iran; 2 Department of Clinical Sciences, School of Veterinary Medicine, Shiraz University, Shiraz, Iran; 3 Department of Clinical Sciences, School of Veterinary Medicine, Islamic Azad University, Kazerun Branch, Kazerun, Iran; 4 Department of Formulation, Barij Medicinal Plants Research Center, Kashan, Iran

**Keywords:** Clinical endometritis, Dairy cow, Penicillin, Streptomycin, *Zataria multiflora*

## Abstract

In the present study, the effect of intrauterine infusion of *Zataria multiflora* extract on the clinical endometritis was investigated. Vaginal examination, transrectal palpation and ultrasonography were used to inspect the genital tract at 30-40 days in milk and two weeks later the same approach was applied. Cows with clinical endometritis were randomly divided into three treatment groups: *Z. multiflora* extract (n = 56), penicillin + streptomycin (pen + strep, n = 55), and placebo (n = 20). Cervical cytology, reagent strip test and cell counting by means of Neubauer hemocytometer were carried out in both examinations. Clinical cure rate of cows with endometritis of score 1 were 45.5, 34.5 and 53.6% in placebo, pen + strep and *Z. multiflora*, respectively. Clinical cure rate of cows with endometritis of score 2, 3 were 66.7, 84.6 and 56.0% in placebo, pen + strep and *Z. multiflora*, respectively. Overall, proportions of successfully treated cows were 55.0, 58.2 and 54.7% in placebo, pen + strep and *Z. multiflora*, respectively (*p* > 0.05). In placebo, none of the parameters were significantly different between first and second examination, while we found the significant differences in percentage of neutrophils and leukocyte esterase activity in other groups (*p *< 0.05). First service conception rate of cows was higher in *Z. multiflora* compared to other groups; however, this difference was not significant. In conclusion, pen + strep and *Z. multiflora* extract can be effective on the clinical endometritis and may improve reproductive performance. The extract of *Z. multiflora* can be useful as an alternative therapy for treatment of clinical endometritis in lactating dairy cows.

## Introduction

Post-partum endometritis is one of the most important problems that reduces reproductive performance in dairy cows.^1^^,^^2^ Endometritisrefers toinfectionorinflammation of the endometrium. It is defined as the presence of purulent (> 50.0% pus) or mucopurulent (approximately 50.0% pus, 50.0% mucus) uterine exudate in the vagina, 21 days or more post-partum in the absence of systemic clinical signs.^3^^,^^4^ Abnormal discharge reflects uterine infection.^5^ Clinical endometritis is diagnosed by transrectal palpation for delayed involution. Transrectal ultrasonography is used to measure the diameter of the uterine horns and cervix, to observe mucus and pus within the uterine lumen, and to examine the contents of the vagina for the presence of pus following manual vaginal examination or vaginoscopes or metricheck.^3^^,^^6^ Leukocyte esterase (LE) is released by neutrophil cells and reacts with indoxil carbonic acid ester. The esterase reaction with diazoniun salt releases indoxil which is oxidized, yielding a violet azo dye.^7^ Recently, in dairy cows, LE test has been proposed as an alternative and creative cow-side test for diagnosing subclinical endometritis. The LE score was correlated with the neutrophil percentage in samples obtained from uterus and cervix.^8^^-^^10^ The percentage of neutrophils in cervical and uterine fluid samples has not been significantly different and cervical sampling was a simple and practical method used in all herds.^11^^,^^12^

Different approaches were used to treat and control endometritis in dairy cows, including intrauterine infusions of antibiotics and/or antiseptics, systemic anti-biotics, and hormonal treatment (e.g. PGF2α). There are alternative therapies which stimulate the natural uterine defense mechanisms. The above mentioned mechanisms include: (i) endotoxins such as lipopolysaccharide of *Escherichia coli*,^13^ (ii) serum, plasma or hyper-immune serum,^14^ (iii) polymorphonuclear leucocyte extracts,^15^ (iv) granulocyte macrophage colony stimulating factors^13^ and herbal medicines.^16^ It has been reported that many of intrauterine infusion of antibiotics have no effect on the reproductive performance and cause microbial resistance against antibacterial drugs. Therefore, many clinicians prefer to use alternative therapies such as herbal medicine.^16^^,^^17^ In human medicine, the use of herbal therapies is outspread^18^ and more than 80.0% of people were treated by herbal medicine in developing countries.^19^ Information about the treatment of reproductive diseases in farm animals with herbal medicine is limited. Nevertheless, herbal medicine had become the subject of recent scientific investigations. In a study by Sarkar *et al*. it was shown that intrauterine infusion of garlic extract was effective for reducing clinical endometritis.^16^ As a therapeutic agent, *Zataria multiflora* has become a subject of recent scientific investigations. *Z. multiflora* is a thyme-like plant that grows wild in central and southern of Iran, Afghanistan, and Pakistan. This plant, also called Shirazian thyme.^20^ Antibacterial, antifungal, antiseptic, analgesic, anti-inflammatory, and antioxidant properties of *Z. multiflora* have been reported.^21^^-^^25^ The main constituents of dried *Z. multiflora* include carvacrol (61.3%) and thymol (25.1%); the main constituents of the fresh plant include thymol (48.4%), carvacrol (12.6%), p-cymene (13.5%), linalool (5.2%), and g-terpinene (3.9%); among them thymol and carvacrol are the most common antimicrobial agents.^19^^,^^26^^,^^27^ Several studies reported the positive effect of *Z. multiflora* for treatment of reproductive disorders such as mycotic vaginitis and bacterial vaginosis in human.^28^^,^^29^ Study of Simbar *et al*. showed that treatment of bacterial vaginosis with *Z. multiflora* vaginal cream was effective with similar results when this disease is treated with metronidazole.^29^ Therapeutic effects of vaginal cream containing garlic and thyme are similar to that of clotrimazole vaginal cream on candida vaginitis.^28^ In a study by Kaveh *et al*. the effects of *Z. multiflora* were investigated in comparison with penicillin + Betadine on improvement of necrotic vaginitis in postpartum dairy cows. They reported that both treatment methods were effective in treating of vaginal necrosis.^30^ No study has been performed to determine the effects of intrauterine infusion of *Z. multiflora* extract on the clinical endometritis in dairy cow. Therefore, the present study was designed to determine the effects of intrauterine infusion of *Z. multiflora* extract on clinical endometritis and reproductive performance in Holstein dairy cows.

## Materials and Methods

**Animals and samples collection. **In the present study, a number of 131 registered primiparous and multiparous Holstein cows from Farzis farm (Milk and Meat Producing Complex in Shiraz, south of Iran) were used. This study was begun in November 2013 and continued until November 2014. The cows were kept under the same management circumstances. All cows were housed in open shed barns, milked three times a day and artificially inseminated exclusively after a voluntary waiting period of 55 days. Cows between 30 to 40 days in milk (DIM) used in the present study. Peri-partum disease, lactation number, parturition status, twin birth and milk production were recorded. Body condition score was recorded at the time of treatment (based on a 1 to 5 score).

Manual vaginal examination, transrectal palpation and ultrasonography were used for inspection of the genital tract. Using manual vaginal examination, the lateral, dorsal and ventral walls of the vaginal fornix and the external cervical os were palpated and the mucus contents withdrawn for examination. Clinical endometritis was confirmed based on the examination of vaginal mucus: score 0 = clear discharge (including both healthy and subclinical endometritis); score 1 = mucus containing flecks of white or off-white pus; score 2 = discharge containing (approximately 50.0% pus, 50.0% mucus material; and score 3 = discharge containing > 50.0% purulent material.^5^

Before the intrauterine infusion, examination of the external genitalia and evaluation of vaginal discharge were performed and the grade of clinical endometritis was categorized. Reproductive organs structure including cervical diameter, position of the uterus, uterine left and right horns diameter and ovaries were evaluated by rectal palpation and ultrasonography. Then, cervical cytology was performed before the intrauterine infusion. Cervical cytology slides were prepared by smearing a drop of cervical mucus on a clean glass microscope slide^11^^,^^12^ and left dried at room temperature and fixed with ethanol for 10 min and stained with a Giemsa stain for 20 min. Cytological assessment was determined based on the percentage of neutrophils by counting 100-200 cells at 400 × magnification in each 20 microscopic field that were randomly selected. Vaginal fornix discharges were collected in 50 mL container and were transferred to laboratory on ice in order to perform the LE test and total white blood cell (t-WBC) count using hemocytometer.

**Reagent test strips and cell counting. **Discharges were strewed on reagent test strips (Analyticon Bio-technologies AG D-35104 Lichtenfels, Germany, Combi screen 11 sys plus). The LE result evaluated based on the color change after 2, 5, 10 and 15 min. The results were categorized in several categories which included as code 0 = negative; code 1 = 25; code 2 = 75 and code 3 = 500 leukocytes per μL with the 0.1 interval based on the color of the reagent area.

A Neubauer hemocytometer was used to manually count t-WBC in the vaginal fornix discharges. Cells were counted on each side of the chamber, averaged, and multiplied by 10 to obtain a total cell count per μL. The cows with clinical endometritis (score 1, 2, and 3) were randomly divided into the following three treatment groups.

**Preparation of** ***Z. multiflora*** **extract and treatment groups. **The hydroalcoholic extract of *Z. multiflora* was processed by Barij Essence Pharmaceutical Company in Kashan, Iran. For the preparation of hydroalcoholic extract (1:2) from *Z. multiflora*, 4.5 kg of dried shoots and powdered plant was mixed with 18 L of 70° ethanol (Barij). The mixture was percolated for 48 hr at room temperature. The extract was then passed through filter paper and the density of extract was determined in the range of 0.918 g per cm. Fifty six cows received intra-uterine infusion of *Z. multiflora* extract (60 mL; 918 mg mL^-1^) and 55 cows received intrauterine infusion of pen + strep (50 mL including 200 + 250 mg mL^-1^; Norbrook Laboratories Ltd., Newry, Northern Ireland), and 20 cows received intra-uterine infusion of placebo (60 mL ethanol 70°). Intrauterine infusion was done by plastic uterine pipette and sterile syringe of 50 mL. All treated cows with clinical endometritis were again examined two weeks later. This was considered as second examination (44 to 54 DIM). We measured the intended parameters before the first and second examinations. The difference value of various parameters was calculated as the value at the first examination minus the value at the second examination.

Clinical cure rate was evaluated through the manual vaginal discharge examination and internal genitalia tract were evaluated as the same manner at the second examination. Then, cervical cytology, t-WBC and LE activity at different times (2, 5 and 10 min after strewing of discharges on the reagent strip) were also performed at second examination. Clinical cure rate was defined as the absence of purulent or muco-purulent discharges and cervical diameter < 7.5 cm at the second examination. Reproductive indices such as calving to first insemination interval, service per conception and days open were recorded and evaluated. First service conception rate (FSCR) was calculated (number of conceived cows divided by total number of first inseminated cows) and considered as an important reproductive index.

**Statistical analysis. **The data was statistically analyzed using the SPSS software (version 15.0, SPSS Inc., Chicago, USA). Cure rate and FSCR of different studied groups were analyzed using the Chi-squared test. Mean (± SE) of percentage of neutrophils, t-WBC and LE activity at different time points of examined cows in both examinations (0 and 14 days) were the subject of comparison between the treatment groups using one way ANOVA followed by LSD, post hoc for multiple comparisons. Reproductive parameters (calving to first service interval, days open and service per conception) were compared between different treatment groups, using Mann Whitney test. Data is presented as the percentage or mean (± SE) and values of *p *≤ 0.05 were considered as significant data.

## Results

 In the present study, 131 dairy cows with clinical endometritis were chosen and three cows were excluded from the study. In this study, prevalence of dairy cows with clinical endometritis of score 1 was 53.1% and the prevalence of dairy cows with endometritis of score 2 and 3 was 46.9%.

Table 1 shows the therapeutic effect of placebo, pen + strep and *Z. multiflora* extract on clinical cure rate in treated cows at the second examination (44 to 54 DIM). In dairy cows with endometritis of score 1, clinical cure rate of studied groups included placebo, pen + strep and *Z. multiflora* extract were 45.5, 34.5 and 53.6% (*p* < 0.05), respectively. Clinical cure rate was 66.7, 84.6 and 56.0% (*p* > 0.05) for placebo, pen + strep and *Z. multiflora* extract groups in dairy cows with endometritis of score 2 and 3.

Overall, proportion of successfully treated cows at the second examination was 55.0, 58.2 and 54.7% in treatment groups of placebo pen + strep and *Z. multiflora *extract, respectively, (*p* > 0.05). In cows with endometritis of score 1, we found the higher cure rate in treated cows with the *Z. multiflora* extract compared to those other treated cows, although this was not significantly different (*p* > 0.05). In cows which were inflicted with endometritis of score 2 and 3, there was significant differences in greater cure rate of treated cows with pen + strep compared to those cows in *Z. multiflora* treated groups (Table 1; *p* < 0.05). Consequently, in cows with different degrees of clinical endometritis, clinical cure rate was not significantly different among groups of placebo, pen + strep and *Z. multiflora* extract (*p* > 0.05).

**Table 1:** Percentages of healthy cows after treatment with placebo, penicillin + streptomycin and *Z. multiflora* extract. Values within the parentheses are the numbers of examined animals

**Groups**	**First examinations**		**Second examinations**	
		**Healthy cows**	**Endometritis (score 1)**	**Endometritis (score 2 and 3)**
***Z. multiflora***	Score 1 (28)	53.6 (15)	32.1 (9)	14.3 (4)
	Score 2 and 3 (25)	56.0 (14) a	0.0 (0)	44 (11)
	Total (53)	54.7 (29)	17 (9)	28.3 (15)
**Pen + strep**	Score 1 (29)	34.5 (10)	58.6 (17)	6.9 (2)
	Score 2 and 3 (26)	84.6 (22) b	0.0 (0)	15.4 (4)
	Total (55)	58.2 (32)	30.9 (17)	10.9 (6)
**Placebo**	Score 1 (11)	45.5 (5)	36.4 (4)	18.2 (2)
	Score 2 and 3 (9)	66.7 (6)	0.0 (0)	33.3 (3)
	Total (20)	55.0 (11)	20 (4)	25 (5)

ab Different superscripts in each column indicate significant differences (*p* < 0.05).

In the first and second examination, we found no significant difference between studied groups in cervical and uterine horns diameter (*p* > 0.05). The means of cervical and uterine horns diameters were 3.3 and 3.5 cm in the first and 3.0 and 3.3 cm in the second examination, respectively. There was no significant difference in the cervical and uterine horns diameters of examined cows between first and second examination.

Percentage of neutrophils, t-WBC (per mL) and LE activity at different time points were not different among treated groups (*p* > 0.05) at first examination (Table 2). The results showed that t-WBC was lower in pen + strep compared to those in placebo and *Z. multiflora* extract groups at the second examination (*p* < 0.05). 

**Table 2:** Mean   SE of percentage of neutrophils, t-WBC and LE activity at different times between treated groups at first (30 to 40 days in milk) and second examination (44 to 54 days in milk

**Examination**	**Groups**	**Neutrophil**	**t-WBC (per L)**	**LE 2**	**LE 5**	**LE10**
**First **	***Z. multiflora***	72.4 ± 4.5	25610.8 ± 3274.5	1.1 ± 0.1	1.8 ± 0.2	2.5 ± 0.2
	**Pen + strep**	79.6 ± 3.6	23290.2 ± 2920.0	1.1 ± 0.1	1.8 ± 0.1	2.6 ± 0.2
	**Placebo**	75.2 ± 7.7	28981.1 ± 7055.4	1.0 ± 0.2	2.0 ± 0.3	2.6 ± 0.3
**Second **	***Z. multiflora***	55.2 ± 5.1	22171.2 ± 3589.9^a^	0.8 ± 0.1 a	1.4 ± 0.2 a	2.0 ± 0.2٭
	**Pen + strep**	50.3 ± 4.3	9905.4 ± 2133.1^b^	0.5 ± 0.1 b	0.9 ± 0.1 b	1.5 ± 0.2٭
	**Placebo**	60.4 ± 10.1	25182.1 ± 5753.8^a^	0.6 ± 0.2	1.2 ± 0.3	1.8 ± 0.3

ab **t-WBC:** Total WBC count of vaginal fornix discharge; **LE 2, 5 and 10:** Leukocyte esterase activity of strip test at time of 2, 5 and 10 min. Different superscripts in each column indicate significant differences (*p* < 0.05);

٭ Indicate tendency to significant differences (*p* = 0.06).

The LE activity of strip test at times of 2 and 5 min was greater (*p* < 0.05) in *Z. multiflora* extract compared to those in pen + strep at the second examination.

In *Z. multiflora* extract group, we found the significant differences in percentage of neutrophils and LE activity at 5 and 10 min; while there was no significant difference in all parameters of placebo group.

The percentages of neutrophils were significantly different between pen + strep and placebo (*p* < 0.05); while, analysis of the other difference values showed no significant difference among the groups (Table 3).

**Table 3:** Difference values (mean ± SE) of parameters during two examinations

**Groups**	**Neutrophil**	**t-WBC (per L)**	**LE 2**	**LE 5**	**LE 10**
***Z. multiflora***	15.7 ± 6.4	3445.5 ± 3682.7	0.3 ± 0.1	0.5 ± 0.2	0.5 ± 0.1
**Pen + strep**	30.1 ± 5.1^a^	12155.4 ± 3048.6	0.6 ± 0.2	0.9 ± 0.2	1.1 ± 0.2
**Placebo**	5.4 ± 10.1^b^	5037.1 ± 9549.7	0.5 ± 0.3	0.8 ± 0.4	0.8 ± 0.5

ab **t-WBC:** Total WBC count of vaginal fornix discharge; **LE 2, 5 and 10:** Leukocyte esterase activity of strip test at time of 2, 5 and 10 min. Different superscripts in columns indicate significant differences (*p* < 0.05).

Table 4 shows reproductive indices of studied cows in placebo, pen + strep and *Z. multiflora* extract groups. The results showed that the first service conception rate of examined cows in *Z. multiflora* extract group was higher compared to those in other groups; however, this difference was not significant (*p* > 0.05). In the present study, there was no significant difference in days open and service per conception indices between studied cows of different treated groups. Calving to first service interval of studied cows in pen + strep was lower compared to those in other groups (*p* < 0.05; Table 4).

**Table 4:** Reproductive indices (mean ± SE) of studied cows in different treated groups

**Groups**	**Calving to 1** ^st^ ** service**	**Days open**	**Service per conception**	**FSCR**
***Z. multiflora***	96.1 ± 4.1 a	133.2 ± 11.2	2.1 ± 0.2	48.9
**Pen + strep**	85.4 ± 2.2 b	124.5 ± 9.8	2.1 ± 0.2	38.8
**Placebo**	98.4 ± 5.1 a	118.0 ± 17.2	1.8 ± 0.2	42.9

**FSCR:** First service conception rate.

ab Different superscripts in columns indicate significant differences (*p* < 0.05).

## Discussion

 The results showed nostatistically significant difference between the effects of various intrauterine infusions used on the clinical endometritis. However, there was a significant difference in greater cure rate of treated cows with pen + strep compared to those cows in *Z. multiflora* treated groups in cows with endometritis of score 2 and 3. We found higher clinical cure rate in treated cows with the extract compared to those other treated cows with endometritis of score 1 though this difference was not significant.

Different approaches were used to treat and control endometritis and numerous studies have been carried out to treat clinical endometritis in dairy cows. A recent review reported that the previous studies showed that PGF_2α_ was not effective against clinical endometritis. Intrauterine infusion of different antibiotics, for example: procaine penicillin, oxytetracycline, ampicillin, cephapirin, ceftiofur were also used for treatment of clinical endometritis.^17^ In others studies aminoglycosides,^31^ chloramphenicol,^32^ sulfonamides^33^ were used for treatment of clinical endometritis. Intrauterine infusion of cephapirin benzathine increased reproductive performance in cow with clinical endometritis and intrauterine infusion of other antibiotics had no effect on improving reproductive performance.^17^ In addition, withdrawal of milk and microbial resistance of antibacterial drugs has been reported after using of antibiotics.^16^ In the study of Sarkar *et al*., the effects of intrauterine infusion of garlic extract and PGF_2α_ injection on the endometritis were investigated. They reported that there was a significant reduction in bacterial load and increasing improvement of reproductive performance in treated cows compared with control group.^16^ Bademkiran *et al*. evaluated the effect of *Pelargonium sidoides* (EPs 7630) on the treatment of clinical endometritis. They reported that there was a significant reduction on days to first service in *P. sidoides* treatment compared with control group.^34^ The *Z. multiflora* is a thyme-like plant. It has antibacterial, antifungal, antiseptic and analgesic, anti-inflammatory, and antioxidant properties.^21^^-^^25^ Previous studies reported effects of *Z. multiflora* on the bacterial growth including some *Bacillus* species,^35^ *Staphylococcus aureus*, *Bacillus subtilis*, *Escherichia coli*,^36^ and *Bacillus cereus*.^37^ The antibacterial effect of *Z. multiflora* is related to components of thymol and carvacrol.^38^

Overall, this study demonstrated that *Z. multiflora* extract has similar effects on improving clinical endometritis compared with treatment group of pen + strep.

Several studies reported the positive effect of *Z. multiflora* for treatment of reproductive disorders.^28^^,^^29^ Simbar *et al*. showed that treatment with *Z. multiflora* vaginal cream was effective and improved the signs and symptoms of bacterial vaginosis and its effect on treatment is similar to that of metronidazole vaginal gel on bacterial vaginosis in human.^29^ Therapeutic effects of vaginal cream containing garlic as same as thyme are similar to clotrimazole vaginal cream on candida vaginitis.^28^

The results of the cure rate of the cows with clinical endometritis of score 1, showed that *Z. multiflora* extract can be more effective on the clinical endometritis in dairy cows. The *Z. multiflora* extract was less effective compared to the pen + strep in clinical endometritis of score 2 and 3. This result showed that pen + strep had more therapeutic effect on severe clinical endometritis. Overall, the results of clinical cure rate showed no significant difference between treatment groups of placebo, pen + strep or *Z. multiflora* extract. This is in agreement with other studies which demonstrated that infusion of penicillin to treat clinical endometritis had no significant difference compared to the untreated control cows.^17^^,^^39^

In previous studies, paraclinical indices including percentage of total neutrophils in cervical cytology,^11^^,^^12^ t-WBC and LE activity^8^^-^^10^ were used for evaluation of treated cows with endometritis. Percentage of neutrophils, t-WBC and LE activity at different time points were not different among treatment groups at first examination. These results demonstrated that the cows in different groups were the same regarding endometritis condition. In the present study, t-WBC of pen + strep group was lower than placebo or *Z. multiflora* extract at the second examination. This is in accordance with the results of clinical cure rate at second examination that was higher for cows that treated with the pen + strep than cows treated with placebo or *Z. multiflora* extract in cows had endometritis of score 2 and 3. These results also showed that t-WBC can be an accurate test for evaluation of endometritis in dairy cows. LE activity of strip test at times of 2 and 5 min was also lower in treatment group of pen + strep than *Z. multiflora* extract group at the second examination. This is in accordance with results of clinical cure rate and t-WBC in cows which had endo-metritis of score 2 and 3.

Changes in percentage of neutrophils in cervical cytology and LE activity at different time points (5 and 10 min) were significantly different from the first and the second examination in treatment groups. These results demonstrated that pen + strep and *Z. multiflora* extract can be used to treat endometritis in dairy cows. Calving to first service interval of studied cows in pen + strep was lower compared to those in other groups. But there was no significant difference in days open and service per conception among studied groups; these results were in agreement with other studies which showed that intra-uterine infusion of penicillin had no effect on reproductive performance.^17^^,^^39^

In the present study, calving to first service interval of studied cows in pen + strep was lower compared to those in other groups. Other reproductive indices were also according to recommended and acceptable range of indices, and no significant difference was found among the groups with this sample size.

In conclusion, pen + strep and *Z. multiflora* extract can be effective on the clinical endometritis and may improve reproductive performance. It is recommended that *Z. multiflora* extract could be considered as an alternative therapy for treatment of clinical endometritis in lactating dairy cows under field condition.
